# Theta and Alpha Alterations in Amnestic Mild Cognitive Impairment in Semantic Go/NoGo Tasks

**DOI:** 10.3389/fnagi.2017.00160

**Published:** 2017-05-23

**Authors:** Lydia T. Nguyen, Raksha A. Mudar, Hsueh-Sheng Chiang, Julie M. Schneider, Mandy J. Maguire, Michael A. Kraut, John Hart

**Affiliations:** ^1^Neuroscience Program, University of Illinois at Urbana-ChampaignChampaign, IL, United States; ^2^Department of Speech and Hearing Science, University of Illinois at Urbana-ChampaignChampaign, IL, United States; ^3^School of Behavioral and Brain Sciences, The University of Texas at DallasRichardson, TX, United States; ^4^Department of Radiology, The Johns Hopkins University School of MedicineBaltimore, MD, United States

**Keywords:** mild cognitive impairment, theta, alpha, Go/NoGo, response inhibition, response execution, cognitive control, categorization

## Abstract

Growing evidence suggests that cognitive control processes are impaired in amnestic mild cognitive impairment (aMCI); however the nature of these alterations needs further examination. The current study examined differences in electroencephalographic theta and alpha power related to cognitive control processes involving response execution and response inhibition in 22 individuals with aMCI and 22 age-, sex-, and education-matched cognitively normal controls. Two Go/NoGo tasks involving semantic categorization were used. In the basic categorization task, Go/NoGo responses were made based on exemplars of a single car (Go) and a single dog (NoGo). In the superordinate categorization task, responses were made based on multiple exemplars of objects (Go) and animals (NoGo). Behavioral data showed that the aMCI group had more false alarms during the NoGo trials compared to controls. The EEG data revealed between group differences related to response type in theta (4–7 Hz) and low-frequency alpha (8–10 Hz) power. In particular, the aMCI group differed from controls in theta power during the NoGo trials at frontal and parietal electrodes, and in low-frequency alpha power during Go trials at parietal electrodes. These results suggest that alterations in theta power converge with behavioral deterioration in response inhibition, whereas alterations in low-frequency alpha power appear to precede behavioral changes in response execution. Both behavioral and electrophysiological correlates combined provide a more comprehensive characterization of cognitive control deficits in aMCI.

## Introduction

Amnestic mild cognitive impairment (aMCI) represents an intermediate stage between normal cognitive aging and dementia in which individuals exhibit a greater decline in cognition than what is expected for their age and education, but is not severe enough to warrant a diagnosis of dementia (Albert et al., 2011; Sperling et al., 2011). It is well recognized that individuals with aMCI are at a higher risk of developing dementia, especially of the Alzheimer's disease (AD) type, compared to cognitively normal older adults (Albert et al., 2011; Petersen, 2011; Jessen et al., 2014). Growing evidence suggests that, in addition to hallmark episodic memory deficits, aMCI individuals experience declines in other cognitive domains, including cognitive control (Traykov et al., 2007; Brandt et al., 2009; Zheng et al., 2012). Cognitive control is essential to our everyday lives as it encompasses a variety of top-down cognitive processes that guide behavior, including response execution and response inhibition (Miyake et al., 2000; Botvinick et al., 2001; Inzlicht et al., 2015). A handful of behavioral studies have noted cognitive control deficits in individuals with mild cognitive decline relative to cognitively normal controls on Erickson flanker (Wylie et al., 2007), Stroop (Traykov et al., 2007; Belanger et al., 2010), Stop-signal (Zheng et al., 2014), and Go/NoGo (Tripathi et al., 2015) tasks, while others have failed to note such deficits (Belleville et al., 2007; Zhang et al., 2007). Given that neuropathological changes associated with dementia are present years before behavioral manifestations (Jack et al., 2013), functional neurocognitive techniques such as electroencephalography (EEG) may add to our understanding of cognitive control deficits in aMCI.

Numerous EEG studies have characterized the effects of normal cognitive aging on cognitive control by examining event-related potentials (ERPs) associated with Go/NoGo tasks (e.g., Pfefferbaum and Ford, 1988; Tachibana et al., 1996; Beste et al., 2010; Vallesi, 2011; Huster et al., 2013; Mudar et al., 2015; Barry et al., 2016; Kropotov et al., 2016), in which participants make a response to certain stimuli (Go) and inhibit a response to other stimuli (NoGo) based on pre-defined criteria. These Go/NoGo studies typically show changes in the N2 and P3 ERP components in normal cognitive aging (e.g., Pfefferbaum et al., 1985; Eimer, 1993; Falkenstein et al., 1999; Mudar et al., 2015; Barry et al., 2016; Kropotov et al., 2016). Only a couple of studies have examined whether aMCI affects ERPs corresponding to Go/NoGo tasks relative to normal cognitive aging (Cid-Fernandez et al., 2014; Mudar et al., 2016). These studies have found that N2 amplitude (Cid-Fernandez et al., 2014) and N2 latency (Mudar et al., 2016) for both Go and NoGo trials differ between the aMCI and control groups, providing preliminary evidence for generalized neural processing alterations related to cognitive control at the aMCI stage. While ERPs provide initial insights into neurophysiological changes related to cognitive control in aMCI, neural oscillations, which provide information about both phase-locked and non-phase-locked activity in the EEG data, can add to our understanding of the changes that occur in the early stages of cognitive decline.

Event-related spectral perturbations (ERSPs), or measures of event-related neural oscillations, allow for analysis of neural responses across multiple frequency bands (e.g., delta, theta, alpha, beta, and gamma bands), each of which have been associated with various cognitive functions (for reviews see Klimesch, 1996, 1999; Basar et al., 2001; Klimesch et al., 2007; Rossini et al., 2007). The theta (4–7 Hz) and alpha (8–13 Hz) bands are particularly relevant to the Go/NoGo paradigm due to their association with cognitive control (Ishii et al., 1999; Yamanaka and Yamamoto, 2010; Nigbur et al., 2011; for reviews see Klimesch, 1996, 1999; Klimesch et al., 2007; Huster et al., 2013; Cavanagh and Frank, 2014; Cavanagh and Shackman, 2015). Studies with cognitively normal individuals have consistently found higher frontal midline theta power for NoGo trials compared to Go trials, leading to suggestions that theta modulations are critical for the recruitment of cognitive control processes linked to response execution and inhibition (Nigbur et al., 2011; Cohen and Donner, 2013; for reviews see Cavanagh and Frank, 2014; Cavanagh and Shackman, 2015). Similarly, studies have shown an association between higher alpha power and active inhibitory mechanisms requiring suppression of distracting or irrelevant information (Cooper et al., 2003; Sadaghiani et al., 2012; Park et al., 2014; Sadaghiani and Kleinschmidt, 2016) in both cognitively normal individuals (for reviews see Klimesch et al., 2007; Jensen and Mazaheri, 2010; Mathewson et al., 2011) and clinical populations (e.g., Roche et al., 2004; Pandey et al., 2016).

Despite the inherent value of ERSPs in characterizing cognitive control processes in real time, there are a limited number of studies that have examined ERSPs in the aMCI population (for a review see Basar et al., 2013). Rather, much of what we know about neural oscillations in aMCI comes from a large body of literature on resting state oscillations (e.g., Rossini et al., 2006; Moretti et al., 2007; Babiloni et al., 2009; for reviews see Babiloni et al., 2011; Moretti et al., 2011). To the best of our knowledge, no studies have examined theta and alpha power corresponding to Go/NoGo tasks in individuals with aMCI. Thus, the goal of the current study was to examine differences between aMCI individuals and cognitively normal controls in theta and alpha band power corresponding to Go/NoGo tasks with varying levels of semantic categorization (basic and superordinate categorization). We hypothesized that overall theta and alpha band power would be attenuated in the aMCI group compared to controls, particularly for the inhibition (NoGo) trials in the more complex superordinate categorization task.

## Materials and methods

### Participants

Twenty-two individuals with aMCI (14 females; mean age = 68.68 years, *SD* = 7.69) and 22 age-, sex-, and education-matched cognitively normal controls (16 females; mean age = 65.32 years, *SD* = 6.84) participated in the study. All participants were 54 years or older, native speakers of English, and had no history of learning disabilities, stroke, major psychiatric illnesses, alcohol or substance abuse, elevated depressive symptoms [Beck Depression Inventory-II (Beck, 1996) or Geriatric Depression Scale > 10 (Almeida and Almeida, 1999)], or uncorrected hearing or vision loss. All aMCI participants met clinical diagnosis of MCI consistent with the guidelines of the 2011 US National Institute on Aging and Alzheimer's Association workgroup (Albert et al., 2011), including: (a) memory concerns reported by the patient and/or corroborated by a reliable informant, (b) episodic memory impairments verified by objective measures, (c) relative independence in activities of daily living, and (d) did not meet criteria for dementia. Participants in the aMCI group completed the Clinical Dementia Rating (Morris, 1993) and received scores of 0.5. Three of the 22 aMCI patients were taking cholinesterase inhibitors when tested, but were on stabilized doses for at least 3 months. Control participants had no subjective cognitive complaints and performed normally on neuropsychological evaluations. Demographic information and results of the neuropsychological assessments for both groups are reported in Table 1. Written informed consent was obtained from all participants in accordance with the protocols approved by the Institutional Review Boards of The University of Texas at Dallas and The University of Texas Southwestern Medical Center. Experiments were performed in accordance with the ethical standards of the Committee on Human Experimentation of these institutions and with the Helsinki Declaration of 1975.

**Table 1:** **Demographics and neuropsychological measures**.

	**Controls**	**aMCI**	***p*-value**
**DEMOGRAPHICS**			
Total N	22	22	–
Age	65.32 (6.84)	68.68 (7.69)	0.133
Education	16.59 (1.65)	16.23 (1.82)	0.492
Sex	16F/6M	14F/8M	0.529
**NEUROPSYCHOLOGICAL MEASURES**			
MMSE^a^	28.75 (0.50)	28.32 (1.29)	0.520
MoCA^b^	28.11 (1.28)	–	–
COWAT - Letter Fluency^c^	44.22 (3.31)	39.36 (11.55)	0.228
Category Fluency^c^	22.22 (2.77)	20.91 (6.28)	0.554
DS Forward	7.27 (1.12)	7.36 (2.65)	0.883
DS Backward	5.45 (1.60)	5.27 (0.94)	0.647
Similarities^d^	26.36 (3.30)	26.10 (3.24)	0.789
TMT-A time^d^	29.00 (9.15)	35.29 (13.22)	0.076
TMT-B time^d^	61.68 (18.57)	85.38 (30.43)	0.004^**^
LM Immediate^d^	16.14 (3.68)	12.05 (3.98)	0.001^**^
LM Delayed^d^	14.50 (3.79)	9.62 (3.25)	<0.001^**^

*Each cell represents group mean (standard deviation)*.

a Controls, n = 4;

b Controls, n = 18;

c Controls, n = 9;

d *aMCI, n = 21*.

*MMSE: Mini-Mental State Exam (Folstein et al., 1975); MoCA, Montreal Cognitive Assessment (Nasreddine et al., 2005); COWAT, Controlled Oral Word Association Test (Benton and Hamsher, 1976); Category fluency (Goodglass et al., 2001); DS, Digit Span (Wechsler, 2008); Similarities (Wechsler, 2008); TMT, Trail Making Test (Reitan, 1958); LM, Logical Memory (Wechsler, 1997). The numbers in the table reflect raw test scores*.

** *p < 0.01*.

### Experimental paradigm and procedures

Participants in both groups completed two visual Go/NoGo tasks involving basic and superordinate categorization during which EEG data were acquired. Go trials required a button press response and NoGo trials required inhibiting/withholding a button press response. The two Go/NoGo tasks were approximately 7 min each and were completed during a single visit with a short break between tasks. These two tasks have been used previously in studies involving younger and older adults (Maguire et al., 2009, 2011; Brier et al., 2010; Mudar et al., 2015), as well as clinical populations with cognitive impairment (Tillman et al., 2010; Mudar et al., 2016). The details regarding the development of these tasks can be found in Maguire et al. (Maguire et al., 2009).

In the single-car task, a basic categorization task, the Go stimulus was a line drawing of a car and the NoGo stimulus was a line drawing of a dog. The images of the car and the dog were presented 160 and 40 times, respectively. The basic-level labels of “car” and “dog” were used to prompt correct discrimination using basic classification (car vs. dog) instead of superordinate classification (vehicle vs. animal). The following instructions were given to the participants: “You are going to see some dogs and cars. When you see a dog, do not push the button. Press the button for anything that is not a dog. Be as quick and as accurate as possible.”

In the object-animal task, a superordinate categorization task, the Go stimuli were line drawings of objects and the NoGo stimuli were line drawings of animals. The object images consisted of 160 different exemplars (40 food items, 40 cars, 20 clothing items, 20 kitchen items, 20 human body parts, and 20 tools), and the animal images consisted of 40 different exemplars of varying visual typicality (e.g., cat, snake, butterfly, lobster). Each of these images were presented once during the task. Participants were given the following instructions: “You are going to see some objects and animals. When you see an animal, do not push the button. Press the button for anything that is not an animal. Be as quick and as accurate as possible.”

Each of the two tasks consisted of 200 stimuli which were black line drawings fitted to a white 600 × 600 pixel square. Of these 200 stimuli, there were 160 (80%) Go trials and 40 (20%) NoGo trials. This trial distribution was used in order to accentuate the tendency for pre-potent responses. Each stimulus was presented for 300 ms followed by a 1,700 ms fixation period (with “+” presented in the center of the display). To minimize order or practice effects, the sequence of the stimuli in each task was pseudo-randomized and the task order was counterbalanced for each participant. A button box was used to register Go responses and record reaction times (RTs).

### EEG data acquisition and processing

Continuous EEG data were recorded using a 64-electrode elastic cap (Neuroscan Quickcap) and a Neuroscan SynAmp2 amplifier and Scan 4.5 software (sampling rate: 1 kHz, DC-200 Hz), with electrode impedances typically below 10 kΩ. The reference electrode was located at the midline between Cz and CPz. Vertical electroocculogram (VEOG) was recorded at sites above and below the left eye. The raw EEG data were processed offline to correct for eye blinks and muscular artifacts using Neuroscan Edit software. Poorly functioning electrodes were identified by visual inspection and excluded from analysis (4.1% in controls and 3.5% in aMCI participants). The continuous EEG data were high-pass filtered at 0.15 Hz and corrected for eye blinks using spatial filtering in Neuroscan. The EEG data were epoched from 500 ms before the onset of the stimuli to 2,000 ms after the presentation of the stimuli. Epochs with peak signal amplitudes of more than 75 μV were rejected. The rejection rates for control/aMCI participants were 9.7/8.0% in Go trials and 8.3/6.8% in NoGo trials. *Post-hoc* analyses did not reveal any significant differences in the rejection rates between groups (*p* > 0.05). Only trials to which the participant responded correctly and those without artifacts were included in the analysis. The EEG data were re-referenced to the average potential over the entire scalp. An algorithm computing the average based on spherical splines fitted to the data (described in Ferree et al., 2009) was then applied to interpolate EEG data to the sites of the bad electrodes.

### EEG spectral analysis

ERSPs were estimated using the EEGLAB toolbox (Delorme and Makeig, 2004) running under Matlab 2013b (MathWorks, Natick, MA, USA) for each group (Controls/aMCI) across the tasks (single-car/object-animal) and response types (Go/NoGo). Epochs were divided into 200 time points between −244 and 1,744 ms. The epochs were processed using a sub-window of 512 ms sliding in 10 ms steps and were zero-padded with a pad-ratio of 2, resulting in an interpolated frequency resolution of approximately 1 Hz per frequency bin. Baseline correction was done in accordance with a gain model (Delorme and Makeig, 2004; Grandchamp and Delorme, 2011), where each time-frequency post-stimulus time point was divided by the average pre-stimulus baseline power at the same frequency. Each sub-window was short time Fourier transformed with Hanning window tapering. The theta and alpha frequency ranges were subsequently analyzed given that studies have associated them with cognitive control and semantic processing (for reviews see Klimesch, 1996, 1999; Klimesch et al., 2007; Huster et al., 2013; Cavanagh and Frank, 2014; Cavanagh and Shackman, 2015).

### Power estimation

We estimated power in the theta (4–7 Hz), low-frequency alpha (8–10 Hz), and high-frequency alpha (11–13 Hz) bands. The frequency ranges of these bands were chosen based on previous ERSP studies (i) involving individuals with MCI (e.g., Jelic et al., 2000; Grunwald et al., 2002; Cantero et al., 2009; Deiber et al., 2009; for review see Drago et al., 2011) and (ii) those that have used cognitive control paradigms (e.g., Brier et al., 2010; Yamanaka and Yamamoto, 2010; Nigbur et al., 2011; Cavanagh and Frank, 2014; Cavanagh and Shackman, 2015). We used traditional alpha bands, as opposed to bands determined by individual alpha frequency (IAF), because we did not observe any differences between groups when IAF was calculated (see Supplementary Materials for details on IAF, including computation, analysis, and results). Peak power was computed for each task (single-car/object-animal) and response type (Go/NoGo) from 0 to 600 ms at three electrode clusters: frontal (Fz, F1, and F2), central (Cz, C1, and C2), and parietal (Pz, P1, and P2). The time period of 0–600 ms was used based on (i) the consistent findings of frontal theta power differences between NoGo and Go trials in this time period by previous studies (e.g., Yamanaka and Yamamoto, 2010; for review see Huster et al., 2013), and (ii) the correspondence of this time period to the time frames of the N2 and P3 ERP components that are typically elicited in Go/NoGo paradigms (e.g., Pfefferbaum et al., 1985; Kok, 1986; Falkenstein et al., 1999; Mudar et al., 2015, 2016; Barry et al., 2016; Kropotov et al., 2016). The electrode clusters were chosen based on (i) the relationship between cognitive control and frontal theta and alpha (for reviews see Mathewson et al., 2011; Huster et al., 2013), (ii) modulations of central and posterior alpha activity in Go/NoGo paradigms (Jensen and Mazaheri, 2010), and (iii) the use of these regions in other theta and alpha studies with clinical populations (e.g., Jelic et al., 2000; Roche et al., 2004; Jiang, 2005; Zheng et al., 2007; Deiber et al., 2009; Caravaglios et al., 2013, 2015; Pandey et al., 2016).

### Statistical analysis

We used standard general linear models (GLMs) to examine behavioral data (RT and error rate) and EEG measures (theta and alpha power). SAS 9.4 (SAS Institute, Cary, NC, USA) was used to evaluate the GLMs, employing the mixed model procedure with the Kenward-Rogers degree of freedom method and default residual maximum likelihood estimation of variance components. The GLMs included group (Controls/aMCI) as a between-subject variable, task (single-car/object-animal) and response type (Go/NoGo) as within-subject variables, and subject as a random term to account for between- and within-subject sources of error variability. As RT data involved only Go trials, the GLM applied to RT entailed only group and task effects and their interactions. Two types of error rates were examined: (1) *misses*, which involved missing the button press for the Go trials, and (2) *false alarms*, which involved a failure in inhibiting the button press for the NoGo trials. EEG data was examined in theta, low-frequency alpha, and high-frequency alpha bands at the three electrode clusters (frontal, central, and parietal). Due to the unequal number of Go and NoGo trials (160 and 40 trials, respectively), we employed weights in the GLMs for the EEG measures to take into account the unequal variances of each subjects' measured responses for each task (single-car/object-animal) and response type (Go/ NoGo). Weights were determined by the number of trials used for the calculation of each EEG measure separately for each subject and trial type, including Go and NoGo trials for both the single-car and the object-animal tasks. Bonferroni corrections were used to correct for multiple comparisons. *P*-values reported in the Results section are significant effects derived from *F-* and *t*-statistics of contrasts of experimental factor means, including interaction contrasts.

## Results

### Behavioral data

Group means for Go-RTs and Go and NoGo error rates across the single-car and object-animal tasks are reported in Table 2.

**Table 2:** **Group means for behavioral data corresponding to the Go/NoGo task**.

	**Controls**	**aMCI**
**SINGLE-CAR TASK**		
Go RT (ms)	361 (60)	375 (74)
Misses (%)	4.3 (6.7)	0.7 (1.5)
False alarms (%)	8.8 (6.3)	15.0 (12.7)
**OBJECT-ANIMAL TASK**		
Go RT (ms)	448 (65)	475 (93)
Misses (%)	5.4 (5.5)	5.1 (7.3)
False alarms (%)	10.0 (8.8)	13.8 (9.0)

*Each cell represents group mean (standard deviation). RT, reaction time; Misses, errors in Go trials; False alarms, errors in NoGo trials*.

#### Reaction times

For Go-RTs, a main effect of task was observed, *F*_(1, 42)_ = 141.22, *p* < 0.001, with significantly longer RTs for the object-animal task (*M* = 461 ms) compared to the single-car task (*M* = 368 ms). No other effects were significant (*p* > 0.05).

#### Error rates

A significant main effect of response type was observed, *F*_(1, 126)_ = 54.97, *p* < 0.001, with more false alarms (i.e., a response to a NoGo stimulus; 11.9%) compared to misses (i.e., lack of response to a Go stimulus; 3.9%); however a significant interaction between group and response type, *F*_(1, 126)_ = 10.33, *p* = 0.002, was also observed. *Post-hoc* analysis revealed higher false alarm rates, *t*_(42)_ = 2.085, *p* = 0.043, in the aMCI group (14.4%) compared to the control group (9.4%), but the groups did not differ on the number of misses, *t*_(42)_ = −1.62, *p* = 0.112. No other effects were significant (*p* > 0.05).

### EEG power

Group means for theta, low-frequency alpha, and high-frequency alpha band power at the three electrode clusters are reported in Table 3. *F*-test results are reported in Table 4.

**Table 3:** **Group means for theta and alpha band power (dB)**.

	**Theta**		**Low-frequency alpha**		**High-frequency alpha**	
	**Controls**	**aMCI**	**Controls**	**aMCI**	**Controls**	**aMCI**
**FRONTAL**						
Single-car Go	4.53 (2.16)	4.60 (2.19)	−3.10 (2.01)	−2.67 (2.19)	−3.18 (1.82)	−2.88 (1.97)
Single-car NoGo	6.61 (4.34)	5.54 (2.91)	−4.10 (2.38)	−4.38 (2.53)	−4.20 (2.16)	−4.22 (2.14)
Object-animal Go	4.81 (2.32)	4.65 (2.36)	−3.53 (2.53)	−3.05 (2.12)	−3.67 (2.43)	−3.29 (1.86)
Object-animal NoGo	6.00 (2.24)	5.24 (2.69)	−3.70 (2.14)	−4.08 (2.82)	−4.17 (2.11)	−4.36 (2.33)
**CENTRAL**						
Single-car Go	3.94 (2.31)	3.81 (2.02)	−3.44 (2.53)	−2.79 (1.83)	−3.52 (2.24)	−2.89 (1.69)
Single-car NoGo	5.84 (4.11)	5.30 (3.18)	−4.52 (2.35)	−3.92 (2.63)	−4.29 (2.19)	−3.80 (2.40)
Object-animal Go	3.76 (2.09)	4.33 (2.37)	−3.58 (2.24)	−2.74 (1.88)	−3.65 (2.09)	−2.93 (1.81)
Object-animal NoGo	5.44 (2.20)	5.80 (2.42)	−4.07 (2.17)	−3.72 (2.25)	−4.23 (2.19)	−3.81 (2.07)
**PARIETAL**						
Single-car Go	4.33 (2.68)	3.68 (2.25)	−3.94 (2.76)	−2.97 (2.54)	−3.97 (2.43)	−3.08 (2.12)
Single-car NoGo	5.61 (3.76)	4.48 (1.84)	−4.94 (3.04)	−4.56 (3.21)	−5.11 (2.50)	−4.42 (2.79)
Object-animal Go	3.98 (2.61)	3.73 (2.54)	−4.30 (3.17)	−3.33 (2.38)	−4.34 (2.88)	−3.32 (2.01)
Object-animal NoGo	5.37 (2.57)	4.09 (2.03)	−4.53 (3.07)	−4.48 (2.79)	−4.96 (2.74)	−4.62 (2.26)

*Each cell represents group mean (standard deviation)*.

**Table 4:** **Statistical results for theta and alpha band power**.

**Effects**	**Theta**	**Low-frequency alpha**	**High-frequency alpha**
**FRONTAL**			
Group	*F*_(1, 478)_ = 0.66, *p* = 0.422	*F*_(1, 478)_ = 0.01, *p* = 0.905	*F*_(1, 478)_ = 0.05, *p* = 0.823
Response type	***F***_**(1, 478)**_ = **43.55**, ***p*** < 0.001^**^	***F***_**(1, 478)**_ = **78.39**, ***p*** < 0.001^**^	***F***_**(1, 478)**_ = **83.41**, ***p*** < 0.001^**^
Task	*F*_(1, 478)_ = 0.77, *p* = 0.382	*F*_(1, 478)_ = 0.20, *p* = 0.652	***F***_**(1, 478)**_ = **6.00**, ***p*** = 0.015^*^
Group × Response type	***F***_**(1, 478)**_ = **5.82**, ***p*** = 0.016^*^	***F***_**(1, 478)**_ = **11.96**, ***p*** = 0.001^**^	***F***_**(1, 478)**_ = **4.87**, ***p*** = 0.028^*^
Group × Task	*F*_(1, 478)_ = 0.11, *p* = 0.736	*F*_(1, 478)_ = 0.22, *p* = 0.643	*F*_(1, 478)_ = 0.14, *p* = 0.712
Response type × Task	***F***_**(1, 478)**_ = **4.00**, ***p*** = 0.046^*^	***F***_**(1, 478)**_ = **11.46**, ***p*** = 0.001^**^	*F*_(1, 478)_ = 3.11, *p* = 0.078
Group × Response type × Task	*F*_(1, 478)_ = 0.69, *p* = 0.408	*F*_(1, 478)_ = 0.38, *p* = 0.538	*F*_(1, 478)_ = 0.38, *p* = 0.536
**CENTRAL**			
Group	*F*_(1, 478)_ = 0.02, *p* = 0.901	*F*_(1, 478)_ = 0.99, *p* = 0.326	*F*_(1, 478)_ = 1.02, *p* = 0.319
Task	*F*_(1, 478)_ = 0.36, *p* = 0.549	*F*_(1, 478)_ = 1.17, *p* = 0.280	*F*_(1, 478)_ = 0.07, *p* = 0.791
Response type	***F***_**(1, 478)**_ = **71.31**, ***p*** < 0.001^**^	***F***_**(1, 478)**_ = **63.59**, ***p*** < 0.001^**^	***F***_**(1, 478)**_ = **49.9**, ***p*** < 0.001^**^
Group × Response type	*F*_(1, 478)_ = 0.69, *p* = 0.405	*F*_(1, 478)_ = 2.16, *p* = 0.142	*F*_(1, 478)_ = 1.45, *p* = 0.229
Group × Task	*F*_(1, 478)_ = 3.82, *p* = 0.051	*F*_(1, 478)_ = 0.23, *p* = 0.629	*F*_(1, 478)_ = 0.07, *p* = 0.797
Response type × Task	*F*_(1, 478)_ = 0.29, *p* = 0.591	*F*_(1, 478)_ = 2.69, *p* = 0.102	*F*_(1, 478)_ = 0.45, *p* = 0.504
Group × Response type × Task	*F*_(1, 478)_ = 0.00, *p* = 0.946	*F*_(1, 478)_ = 0.75, *p* = 0.387	*F*_(1, 478)_ = 0.15, *p* = 0.694
**PARIETAL**			
Group	*F*_(1, 478)_ = 1.83, *p* = 0.183	*F*_(1, 478)_ = 0.58, *p* = 0.451	*F*_(1, 478)_ = 1.17, *p* = 0.285
Response type	***F***_**(1, 478)**_ = **22.72**, ***p*** < 0.001^**^	***F***_**(1, 478)**_ = **75.85**, ***p*** < 0.001^**^	***F***_**(1, 478)**_ = **104.32**, ***p*** < 0.001^**^
Task	*F*_(1, 478)_ = 2.08, *p* = 0.150	*F*_(1, 478)_ = 0.19, *p* = 0.665	*F*_(1, 478)_ = 3.07, *p* = 0.080
Group × Response type	***F***_**(1, 478)**_ = **3.97**, ***p*** = 0.047^*^	***F***_**(1, 478)**_ = **9.75**, ***p*** = 0.002^**^	*F*_(1, 478)_ = 3.81, *p* = 0.052
Group × Task	*F*_(1, 478)_ = 0.20, *p* = 0.659	*F*_(1, 478)_ = 0.61, *p* = 0.437	*F*_(1, 478)_ = 0.38, *p* = 0.540
Response type × Task	*F*_(1, 478)_ = 0.45, *p* = 0.502	***F***_**(1, 478)**_ = **6.68**, ***p*** = 0.010^**^	*F*_(1, 478)_ = 1.37, *p* = 0.242
Group × Response type × Task	*F*_(1, 478)_ = 0.61, *p* = 0.437	*F*_(1, 478)_ = 1.03, *p* = 0.310	*F*_(1, 478)_ = 1.77, *p* = 0.184

*Group, Controls/aMCI; Response type, Go/NoGo; Task, single-car/object-animal. Values in bold represent significant effects*.

* *p < 0.05*,

** *p < 0.01*.

#### Theta (4–7 Hz)

**Figure 1:**
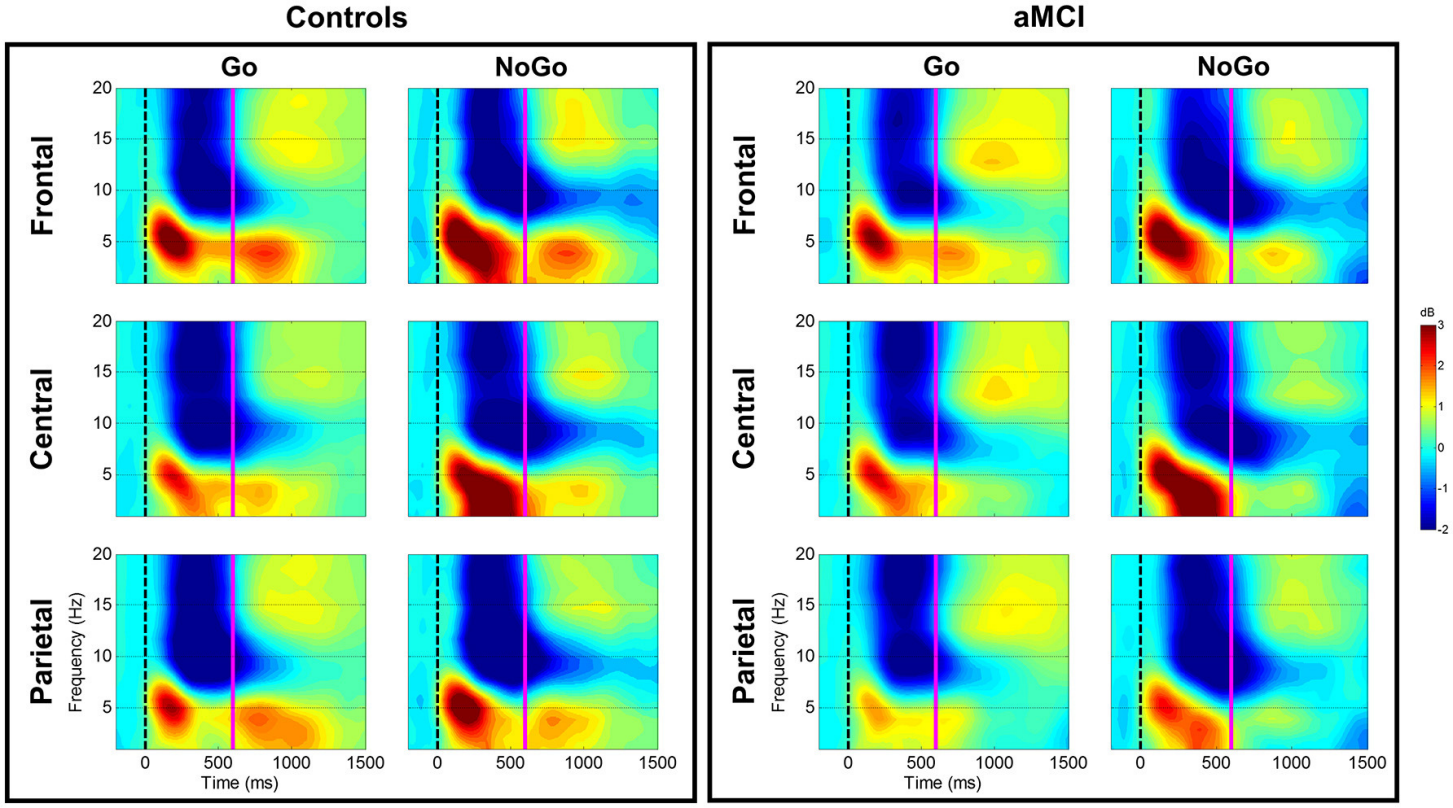
**Group comparison of spectrograms for response types across electrode clusters**. The spectrograms for the Controls and aMCI groups across Go and NoGo trials at the three electrode clusters (Frontal/Central/Parietal) are represented. The zero millisecond (ms) time point (dashed black line) represents the Go or NoGo stimulus onset. Theta (4–7 Hz), low-frequency alpha (8–10 Hz), and high-frequency alpha (11–13 Hz) power were computed between zero ms (dashed black line) and 600 ms (solid magenta line).

**Figure 2:**
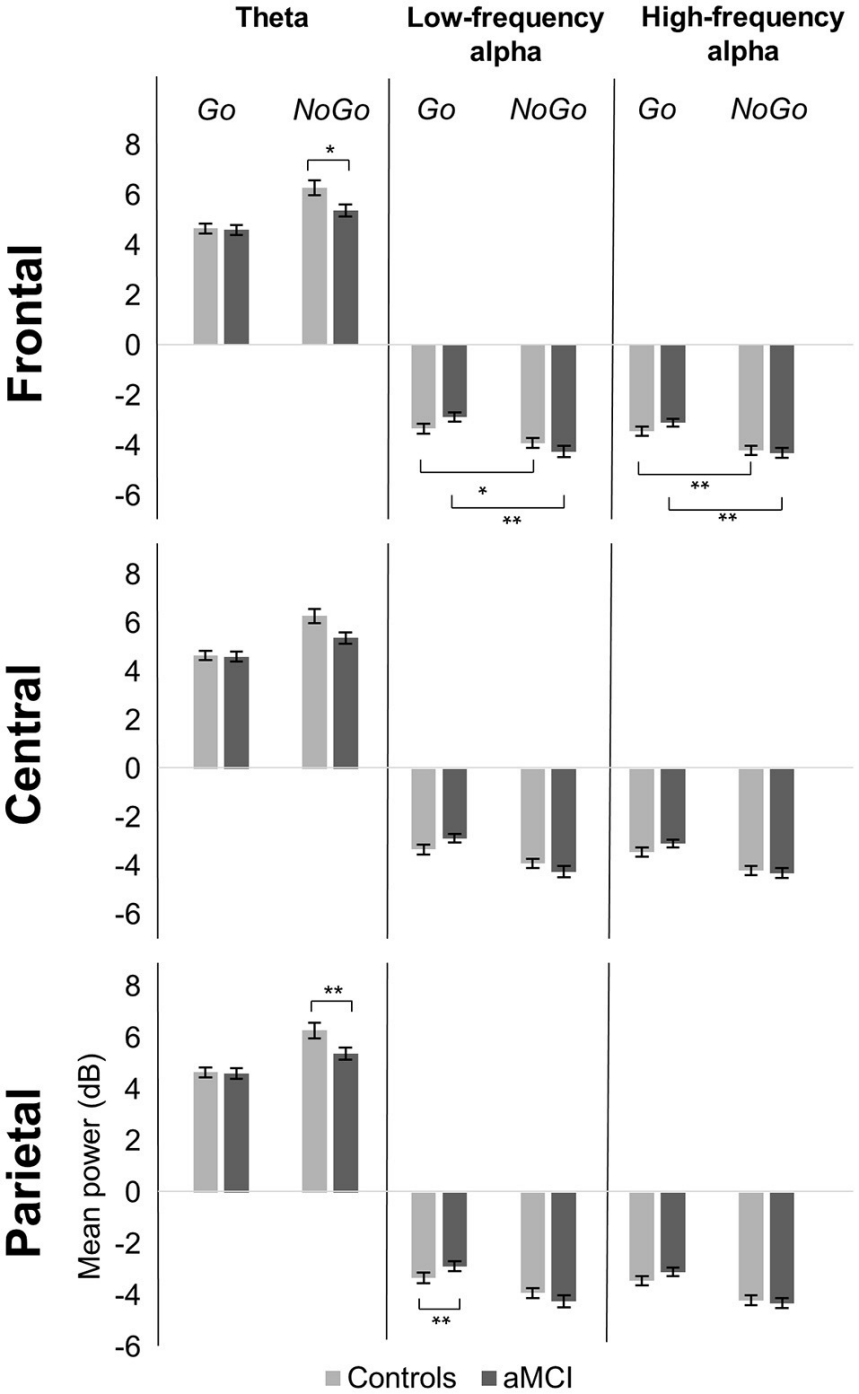
**Group comparison of mean power for response types across electrode clusters**. The bar graphs represent mean power in the theta (4–7 Hz), low-frequency alpha (8–10 Hz), and high-frequency alpha (11–13 Hz) bands for Go and NoGo trials in the Controls and aMCI groups at the three electrode clusters (Frontal/Central/Parietal). Error bars represent standard error. ^*^p < 0.05, ^**^p < 0.01.

Significant main effects of response type were observed at frontal (*p* < 0.001), central (*p* < 0.001), and parietal (*p* < 0.001) electrode clusters with NoGo trials having higher theta power than Go trials (frontal: 5.85 vs. 4.65 dB; central: 5.59 vs. 3.96 dB; parietal: 4.89 vs. 3.93 dB). Furthermore, significant two-way interactions between group and response type were observed in the frontal (*p* = 0.016) and parietal (*p* = 0.047) electrode clusters (Figure 1). These interactions were driven by between group differences in NoGo trials (frontal: *t*_(262)_ = −2.35, *p* = 0.020; parietal: *t*_(262)_ = −3.71, *p* < 0.001), where the control group had higher theta power than the aMCI group (frontal: 6.30 vs. 5.39 dB; parietal: 5.49 vs. 4.28 dB; Figure 2). Additionally, a two-way interaction between task and response type was noted at the frontal electrode cluster (*p* = 0.046), but *post-hoc* tests did not yield any significant differences (*p* > 0.05). There were no other significant results for theta (*p* > 0.05). All statistical results are reported in Table 4.

#### Low-frequency alpha (8–10 Hz)

For low-frequency alpha power, significant main effects of response type were observed at frontal (*p* < 0.001), central (*p* < 0.001), and parietal (*p* < 0.001) electrode clusters with higher power in NoGo trials compared to Go trials (frontal: −4.06 vs. −3.09 dB; central: −4.06 vs. −3.14 dB; parietal: −4.63 vs. −3.64 dB). Significant two-way interactions between group and response type were observed at the frontal (*p* = 0.001) and parietal (*p* = 0.002) electrode clusters (Figure 1). *Post-hoc* tests for the frontal electrode cluster revealed within group alpha power differences between Go and NoGo trials in both the aMCI group, *t*_(262)_ = 4.59, *p* < 0.001, and the control group, *t*_(262)_ = 2.09, *p* = 0.038; however the magnitude of difference was larger in the aMCI group compared to the controls (difference in aMCI of 1.37 dB vs. difference in controls of 0.58 dB). The interaction in the parietal electrode cluster was driven by between group differences in Go trials, *t*_(262)_ = 2.90, *p* = 0.004, where the control group had higher alpha power than the aMCI group (−4.12 vs. −3.15 dB; Figure 2). Significant two-way interaction effects between task and response type were also observed in the frontal (*p* = 0.001) and parietal (*p* = 0.010) electrode clusters, but *post-hoc* tests did not show any significant differences (*p* > 0.05). There were no other significant results for low-frequency alpha (*p* > 0.05). All statistical results are reported in Table 4.

#### High-frequency alpha (11–13 Hz)

For high-frequency alpha power, significant main effects of response type were observed in frontal (*p* < 0.001), central (*p* < 0.001), and parietal (*p* < 0.001) electrode clusters with NoGo trials having higher power than Go trials (frontal: −4.23 vs. −3.25 dB; central: −4.03 vs. −3.25 dB; parietal: −4.78 vs. −3.68 dB). A significant main effect of task was observed in the frontal electrode cluster (*p* = 0.015), with the superordinate categorization (object-animal) task having higher alpha power than the basic categorization (single-car) task (−3.87 vs. −3.62 dB). A significant two-way interaction effect between group and response type was observed in the frontal electrode cluster (*p* = 0.028; Figure 1). *Post-hoc* tests revealed within group alpha power differences between Go and NoGo trials in both the aMCI group, *t*_(262)_ = 4.71, *p* < 0.001, and the control group, *t*_(262)_ = 2.87, *p* = 0.005 (Figure 2); however the magnitude of difference was larger in the aMCI group compared to the controls (difference in aMCI of 1.21 dB vs. difference in controls of 0.75 dB), similar to the findings in low-frequency alpha. There were no other significant results for high-frequency alpha (*p* > 0.05). All statistical results are reported in Table 4.

## Discussion

The current study examined ERSP differences between aMCI and cognitively normal control groups related to response execution and response inhibition using two Go/NoGo tasks that involved semantic categorization. The aMCI group differed from the control group on NoGo trials in false alarm rates and theta power, and on Go trials in low-frequency alpha power. Additionally, neuropsychological measures revealed deficits in the aMCI group relative to the control group on measures of cognitive control (Trail Making Test B) and episodic memory (logical memory immediate and delayed).

Between group ERSP differences related to response type were observed in theta and low-frequency alpha bands in frontal and parietal electrode clusters. Theta power for inhibition (NoGo) trials in frontal and parietal electrode clusters was higher (i.e., more positive) in the control group compared to the aMCI group as hypothesized (Figures 1, 2). Given the relationship between theta band and cognitive control (Ishii et al., 1999; Yamanaka and Yamamoto, 2010; Nigbur et al., 2011; for reviews see Huster et al., 2013; Cavanagh and Frank, 2014; Cavanagh and Shackman, 2015), the NoGo differences observed between the groups seem to indicate that the aMCI group were less able to effectively attend to and suppress their responses for the inhibition trials. Behavioral findings from the current study support this interpretation as the aMCI group showed significantly higher error rates for the NoGo trials, as well as longer Trail Making Test B times, compared to the control group. This finding is in line with studies that have shown higher theta power for cognitively normal controls compared to individuals with MCI (e.g., Missonnier et al., 2006; Caravaglios et al., 2013). The attenuation of theta power observed in the current study can serve as an early objective diagnostic marker of aMCI once this finding is validated by similar studies. Furthermore, such neurocognitive markers would be valuable in evaluating treatment response to novel pharmacological or non-pharmacological interventions in this population.

With regards to the scalp distribution of our theta findings, it is interesting to note that we observed group differences in frontal and parietal electrode clusters, but not the central electrode cluster. Deiber et al. (2007) have noted that theta band shows two topographically separate neural processes, where frontal theta depends on the attentional requirements of the task, and posterior theta relates to stimulus processing without effects of task demands. Given that our paradigm is unable to parse out the attentional requirements from stimulus processing, we are unable to say whether general stimulus processing is impaired in the aMCI group in addition to attentional processing. Examination along these lines with paradigms that can deconstruct these processes are warranted.

Low-frequency alpha band power in the parietal electrode cluster showed between group differences in response execution (Go) trials. As illustrated in Figures 1, 2, the control group had higher alpha band power (i.e., more negative) compared to the aMCI group. Previous studies have suggested a relationship between low-frequency alpha power and attention (for reviews see Klimesch, 1996, 1999; Klimesch et al., 2007). It is likely that the between group low-frequency alpha power difference observed here suggests altered attentional capacity in the aMCI group compared to the control group during Go trials as well. Given that the two groups did not show differences in error rates for Go trials (misses), alterations in low-frequency alpha band power appear to precede behavioral deficits. Similarly, our previous ERP study involving the same Go/NoGo tasks found longer Go-N2 latency for the aMCI group compared to controls without corresponding differences in RTs or error rates (Mudar et al., 2016), indicating that EEG measures may capture early changes in response execution processes in the aMCI population prior to obvious behavioral deficits. Examining these EEG markers in relation to other biomarkers, such as amyloid-based cerebrospinal fluid or fluorodeoxyglucose-positron emission tomography, will further establish their diagnostic utility. In addition to between group differences in alpha power, the aMCI group also showed a greater difference between response execution (Go) and inhibition (NoGo) trials compared to controls in low-frequency alpha power (frontal and parietal electrode clusters) and high-frequency alpha power (frontal electrode cluster). These findings further suggest that the aMCI and control groups differentially allocate resources to discriminate between Go and NoGo trials, supporting alterations in cognitive control in the aMCI group in comparison to cognitively normal controls.

Despite between group differences in theta and low-frequency alpha power, the two groups showed similar effects of response type in all frequency bands examined (theta, low-frequency alpha, and high-frequency alpha) at the three electrode clusters (frontal, central, and parietal), as noted by main effects of response type. In both groups, peak power in all three bands was higher during the inhibition (NoGo) trials compared to the response execution (Go) trials (i.e., more positive theta power and more negative alpha power). Our findings further support the association between response inhibition and higher theta and alpha band power that other studies have observed (e.g., Nigbur et al., 2011; Sadaghiani et al., 2012; Cohen and Donner, 2013; Sadaghiani and Kleinschmidt, 2016; for reviews see Cavanagh and Frank, 2014; Cavanagh and Shackman, 2015). Given that the trial distribution in our study was uneven with frequent Go trials (80%) and infrequent NoGo trials (20%), it could be argued that this finding is related to the processing of less frequent and/or more challenging targets in general rather than being unique to NoGo trials. However, during statistical analysis, models were weighted for number of accepted trials to mitigate this possibility. Thus, this finding provides evidence that both aMCI and cognitively normal individuals engaged more cognitive resources to process the NoGo trials relative to the Go trials. Nonetheless, in light of the between group differences in response type discussed earlier, it appears that cognitive control processes in aMCI are altered beyond what is noted in cognitively normal aging.

Similarities were also observed between the two groups for task effects in high-frequency alpha power in the frontal electrode cluster, where peak power was higher for the superordinate categorization (object-animal) task than the basic categorization (single-car) task. In comparison to basic categorization, superordinate categorization extends beyond perceptual similarities as members of the same superordinate category can share relatively few perceptual features (e.g., the superordinate category “animals” includes both “cat” and “snake”), meaning the object-animal task is more reliant on semantic information (e.g., Large et al., 2004; Maguire et al., 2009) and involves additional neural resources (e.g., Raposo et al., 2012; Chiang et al., 2013). Our finding of higher power in the high-frequency alpha band coincides with studies showing higher alpha power for more complex and/or difficult tasks (Benedek et al., 2011, 2014; for reviews see Klimesch et al., 2007; Jensen and Mazaheri, 2010), as well as those who have shown a relationship between high-frequency alpha band and semantic processing (for reviews see Klimesch, 1996, 1999; Klimesch et al., 2007). Furthermore, this finding aligns with the RT data which showed longer RTs for the superordinate categorization task relative to the basic categorization task, demonstrating that both aMCI and cognitively normal individuals required more effort and processing time for the more semantically complex task. Although we had hypothesized that aMCI individuals would show more pronounced changes for the more complex superordinate categorization task, our results did not support this hypothesis. It is possible that the aMCI individuals in the current study are in the very early stages of cognitive deterioration with relatively preserved semantic processing, as suggested by their category fluency scores (Table 1). Consequently, the complexity of categorization may not have impacted processing in these individuals above and beyond what is typically observed with normal cognitive aging. Incorporating time pressure to respond (i.e., requiring responses to be made within certain reaction time deadlines; Gajewski and Falkenstein, 2013), may have captured the differences across semantic categorization better and needs to be explored in future studies.

In conclusion, individuals with aMCI differed from cognitively normal aging controls on both behavioral (error rates) and EEG measures (theta and low-frequency alpha band power) of cognitive control assessed using two semantic categorization Go/NoGo tasks. While behavioral findings in aMCI largely support impairment on inhibition trials, EEG data suggests that not only does the aMCI group differ from controls in response inhibition, but underlying neurophysiological alterations in response execution are also present. Specifically, our findings indicate that in aMCI, theta power alterations converge with behavioral deterioration in response inhibition, while alterations in low-frequency alpha power precede behavioral changes in response execution. Although our study focused on theta and alpha bands due to their particular relevance to the Go/NoGo paradigm, future studies should examine the utility of other neural oscillations (e.g., delta, beta) in relation to cognitive control paradigms in aMCI to more fully characterize the neurophysiological changes that occur in this population. Given the relationship between cognitive control and independent activities of daily living, identifying behavioral and neural markers related to changes in cognitive control in aMCI has implications for improved characterization of aMCI, early diagnosis, and for evaluating whether novel therapeutic agents positively impact such top-down processing.

## Author contributions

LN processed and analyzed the EEG data, performed statistical analyses, and drafted the manuscript. RM oversaw all aspects of the current study. HC helped with data collection, analysis of the EEG data, and with manuscript preparation. JS helped with the analysis of the EEG data. MM, MK, and JH designed the task and helped with data interpretation and manuscript preparation. All authors read and approved the final manuscript.

## Funding

This work was supported by the Alzheimer's Association New Investigator Grant (NIRG-11-173815); the RGK foundation; and the National Institutes of Health (RC1-AG035954, P30AG12300).

### Conflict of interest statement

The authors declare that the research was conducted in the absence of any commercial or financial relationships that could be construed as a potential conflict of interest.
