# Supplementary material for: Theta and Alpha Alterations in Amnestic Mild Cognitive Impairment in Semantic Go/NoGo Tasks
Source: Front Aging Neurosci. 2017 May 23;9:160. doi: 10.3389/fnagi.2017.00160 (PMC5440918; doi:10.3389/fnagi.2017.00160)
Supplement: Supplementary file 1 [file DataSheet1.PDF]

Supplementary Material

Theta and Alpha Alterations in Amnestic Mild Cognitive Impairment in Semantic Go/NoGo Tasks

Lydia T. Nguyen, Raksha A. Mudar*, Hsueh-Sheng Chiang, Julie M. Schneider, Mandy J. Maguire, Michael A. Kraut, John Hart Jr.

***Correspondence:** Raksha A. Mudar, raksha@illinois.edu

1 Individual alpha frequency (IAF)

For each participant, an average of all the individual electrodes was used to create global power spectra for each task (single-car/object-animal) and condition (Go/NoGo). IAF was determined by identifying the frequency that had peak power within the extended alpha range (7-14 Hz) in the global spectrum [1]. IAF was calculated separately for each task and condition, resulting in four IAF values (single-car Go, single-car NoGo, object-animal Go, and object-animal NoGo). Group means and *p*-values for the IAF values are reported in Table 1. There were no significant group differences for IAF.

Table 1. Group means for individual alpha frequency (Hz). Each cell represents group mean (standard deviation).

	**Controls**	**aMCI**	***p*-value**
Single-car Go	10.03 (2.72)	9.90 (2.96)	.877
Single-car NoGo	9.14 (2.53)	8.48 (1.99)	.337
Object-animal Go	9.68 (2.37)	9.37 (2.95)	.702
Object-animal NoGo	8.92 (2.24)	8.97 (2.50)	.951

Reference

1. Klimesch, W., *EEG alpha and theta oscillations reflect cognitive and memory performance: a review and analysis.* Brain Res Rev, 1999. **29**(2-3): p. 169-95.
